# OpenMCT: an open-source DC motor control educational kit

**DOI:** 10.1016/j.ohx.2026.e00794

**Published:** 2026-05-22

**Authors:** Alejandro Von Chong, Salvador Vargas, Jean-François Duhé, Dorindo Cárdenas

**Affiliations:** a School of Electrical Engineering, Universidad Tecnológica de Panamá, Víctor Levi Sasso Campus, Panama City, Panama; b Facultad de Informática, Electrónica y Comunicaciones, Universidad de Panamá, Panama City, Panama; c Sistema Nacional de Investigación-SENACYT, Panama City, Panama

**Keywords:** DC motor, Control theory, Educational hardware

## Abstract

Control engineering is used in multiple engineering domains, but most introductory courses lean heavily on mathematical manipulation and simulation. This can limit students’ exposure to real-world applications, often at the cost of student engagement. To promote a system-level approach, this work describes OpenMCT, a low-cost, open-source educational kit for DC motor control that enables hands-on experiments covering topics from actuation and sensing to sampling and real-time control execution, while also touching on other engineering topics students encounter in practice, such as drive electronics, measurement conditioning, and filtering. The hardware integrates a brushed DC motor, incremental encoder feedback, motor current sensing, and an H-bridge driver controlled by a Teensy 4 microcontroller. A cross-platform Python/Qt GUI provides configuration, live telemetry, excitation signals, data logging, and interactive controller tuning, including PID and direct entry of z-domain difference-equation coefficients. Validation follows the workflow commonly taught in control courses, going from calibration, characterization, and system identification through to controller design and deployment. Experimental results show that system identification and closed-loop responses follow the trends predicted by the GUI-based analysis and simulations.

## Graphical abstract

**Figure:**
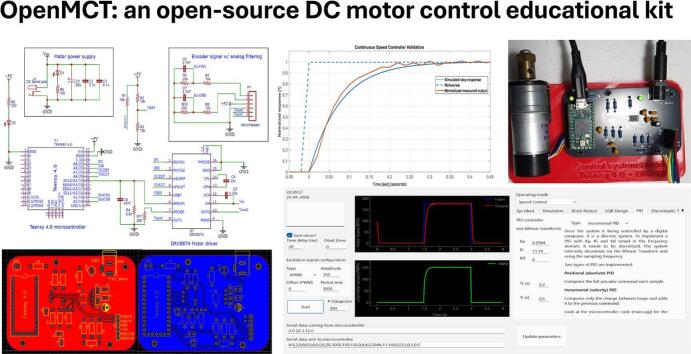

**Specifications table**

**Table:** 

Hardware name	OpenMCT
Subject area	• Engineering and materials science • Educational tools and open-source alternatives to existing infrastructure
Hardware type	• Electrical engineering and computer science
Closest commercial analog	QNET 2.0 DC Motor Board for NI ELVIS
Open source license	CERN-OHL-P-2.0
Cost of hardware	∼USD 100
Source file repository	https://zenodo.org/records/19986592
OSHWA certification UID	PA000005

## 1 Hardware in context

Engineering is a problem-solving profession that requires a problem-based approach to learning [1]. This profession is of vital importance to achieving the United Nations’ 17 sustainable development goals and thus, to the future of the planet [1], [2]. In engineering education, significant challenges continue to be faced, primarily related to providing new methods for specialized training in a domain where problems are no longer confined to an individual discipline [3]. As time passes, technology is becoming more complex and the learning outcomes for engineering must follow suit, so future engineers should master technical concepts and be able to identify and solve complex, real-world problems [1], [4], [5].

In the past, most control engineering courses would likely have been focused on theoretical, abstract aspects and simulations, but programming and implementation were rarely included [6]. As a result, many students would complete a first control course but never really appreciate what control is nor why it is important. Even if mathematical rigor is important, there comes a point where pen and paper computations are a barrier to efficient progress and understanding because the process is too slow. Letting a computer do the heavy math is an obvious way to overcome this [7], [8]. Once students have mastered the core concepts, it makes more sense to let computers handle calculations so students can focus more on interpretation, analysis, evaluation, and design.

The practical application of control requires an understanding of both theoretical principles and real-world system behaviors. Thus, in the context of teaching students the skills needed to solve real-world problems, control can play an important role because it naturally takes a multidisciplinary perspective and has a wide variety of applications [4]. Implementation of control systems is gaining importance because of the industries’ demands, which result from a dramatic growth in technological capabilities that require a tight interaction between control and computer science [6].

To this end, a variety of educational hardware implementations proposed in the literature are hardware specifically designed for control teaching (e.g. MotoShield and the Arduino Moto Shield v3) [5], [9], [10], [11], [12], [13], [14], [15], [16], [17], while others focus on virtual and remote laboratories [7], [18]. Additionally, there is commercially available hardware for control teaching, such as Quanser’s Qube-Servo 3 [19], Edibon’s DC Servo Motor Module [20], and Festo’s Process Control Learning System [21]. These platforms provide valuable learning experiences, but they often involve higher acquisition costs, proprietary software dependencies, reduced hardware-level accessibility, and limited freedom for long-term modification or repair. In parallel, growing interest has emerged in low-cost and take-home laboratory platforms that can be fabricated, assembled, and modified using widely available components [22], [23], [24]. Such platforms aim to increase accessibility, promote active learning, and provide continuity between theoretical instruction and practical experimentation.

In this context, the platform presented in this work contributes an open-source, low-cost, and reproducible DC motor control kit that integrates the complete experimental workflow within a single environment. The proposed system combines fabrication files, embedded firmware, and a cross-platform Python/Qt GUI so that users can move from assembly and calibration to plant characterization, system identification, controller tuning and design, implementation, and experimental validation on the same hardware. Its educational value lies in making the digital control workflow directly observable and modifiable, including actuator commands, sampled measurements, fitted plant models, and controller coefficients executed on the microcontroller. This makes the platform suitable for hands-on experimentation in introductory control courses, customization, and long-term reuse in open laboratory settings.

## 2 Hardware description

The hardware presented in this work is an open-source educational platform designed specifically for practical teaching and experimentation in control theory. Unlike existing educational control hardware, which often relies on proprietary third-party software (e.g. MATLAB/Simulink and LabVIEW), proprietary DAQ units, and fixed configurations, this platform integrates widely available, low-cost components and fully open-source software environments. The hardware is used in conjunction with a visualisation/control software which uses SciPy and Python-Control, both robust, open-source Python libraries recognized in the scientific community and used in a wide range of applications [25], [26], [27], [28]. SciPy provides a suite of tools for numerical computation, optimization, and signal processing, whereas Python-Control is focused on classical and modern control theory [29]. Compared with proprietary environments such as MATLAB, these libraries provide a broad set of numerical and control-analysis functions without licensing costs. Broadly speaking, the proposed system covers the following aspects:

• Supports hands-on assembly (mostly through-hole soldering) and an end-to-end build–test workflow, rather than only interacting with preassembled didactic trainers.
• Enables open-loop and closed-loop experiences on a DC motor with encoder (speed and position), with real-time plotting and data logging.
• Covers static nonlinearity characterization, data acquisition, empirical model fitting, simulation, controller design, and experimental validation within a single platform.
• Allows users to implement arbitrary discrete-time controllers, including recursive (IIR) structures, by directly entering difference-equation (z-domain) coefficients, not only predefined PID blocks.
• The GUI is protocol-driven and can be adapted to other plants by streaming the expected variables in the required order and with consistent scaling.

The hardware comprises a brushed DC motor with quadrature encoder feedback and a DRV8874 H-bridge motor driver. The motor is controlled via a Teensy 4.0, providing flexibility in computational performance and feature expansion. A Python-based graphical user interface (GUI) developed with Qt complements the hardware, providing real-time visualization, interactive parameter tuning, and data logging. This allows students and educators to implement and observe a variety of control strategies, extending beyond basic PID experimentation. The proposed system supports a complete experimental workflow in control engineering, including calibration, system identification, time-response simulation, continuous- and discrete-time control analysis, controller tuning and design, plant discretization, implementation of digital controllers via difference equations, and experimental validation on the same hardware platform.

Commercial educational platforms for control theory teaching, such as Quanser’s Qube-Servo 3, NI ELVIS III, and dSPACE MicroLabBox, support controller design and implementation through graphical environments, model-based workflows, and automated deployment tools. In the proposed platform, users can also work directly with the coefficients of discrete-time controllers, making it possible to implement and test z-domain control laws through explicit difference-equation representations on the embedded hardware. This complements higher-level analysis and visualization tools by exposing the sampled-data controller structure more directly for teaching purposes.

Also, unlike many commercial solutions (e.g., Quanser Qube-Servo 3, NI ELVIS, dSPACE, Festo Didactic, OPAL-RT), this hardware is open and modifiable. Commercial alternatives, while robust, typically have high initial costs, recurring software licensing fees, limited modularity, and a significant risk of vendor lock-in and eventual obsolescence. For instance, platforms such as the QNET 2.0 DC Motor Board for NI ELVIS have already faced discontinuation, creating challenges regarding support, upgrading paths, and component replacement (see Table 1). Conversely, the proposed open-source design significantly reduces these risks by utilizing non-specialized components available from mainstream electronics distributors, thus ensuring long-term availability and repairability.Table 2.

**Table 1:** Comparison between the proposed and commercially available equipment for Control Systems Teaching.

**Feature**	**Commercial Systems (Quanser, NI, dSPACE, Festo, OPAL-RT)**	**Proposed Platform**
Software dependence	Proprietary (MATLAB/Simulink, LabVIEW) with ongoing licensing fees and compatibility constraints	Fully open-source (C++, Python)
Cost per unit	Typically USD 3,000–20,000 + per system	< USD 100 using widely accessible parts (considering the BOM cost).
Customization	Limited: expansions are costly, require expensive vendor-specific modules. Some expansions/modules are inexistent.	Fully customizable: open access to all hardware and software. Inexpensive integration of sensors, actuators or alternative controllers
Discontinuation risk	Moderate to high: discontinuation can lead to loss of support and costly replacements (e.g., NI ELVIS III)	Very low: widely supported open-source hardware and software ecosystems

**Table 2:** Commercially available components for the proposed device.

**Component**	**Processing Power**	**Real-time Capability**	**Logic Level Compatibility**	**Efficiency**	**Integrated Protection**	**Cost Effectiveness**	**Ease of Setup**
**Microcontroller**							
Teensy 4.0 (**selected**)	Very High	Very High	High	N/A	N/A	Good	Good
Arduino Uno	Low	Average	Average	N/A	N/A	Very Good	Very Good
Raspberry Pi 5	Extremely High	Low	High	N/A	N/A	Average	Average
**Motor Driver**							
DRV8874 (**selected**)	N/A	High	Very High	Very High	Very High	Good	Very low
L298N	N/A	Average	Low	Low	Low	Very Good	Very Good
TB6612FNG	N/A	High	High	High	Average	Good	Good

The hardware proposed in this work comprises hardware and software implementations, which are described in the following sections.

### 2.1 DC motor module

The DC motor module consists mainly of a microcontroller unit, a motor driver and a brushed DC motor. For the microcontroller unit, the Teensy 4.0 (SparkFun Electronics, Niwot, CO, USA) was chosen primarily due to its high computational capacity (600 MHz processor) and real-time processing capability. While the current implementation is based on PID and difference-equation-based controllers (which are not computationally expensive), its high processing power ensures flexibility for more computationally demanding controllers for future system expansion, allowing scalability and the implementation of more sophisticated control algorithms. Furthermore, the Teensy 4.0 is compatible with popular programming IDEs, such as Arduino IDE and Visual Studio Code.

For the motor driver, a DRV8874 (Texas Instruments Inc., Dallas, TX, USA) was selected. It integrates an N-channel MOSFET H-bridge and a charge pump that enables 100% duty cycle operation [30]. Despite being less popular than other commonly used motor drivers, the DRV8874 provides several notable advantages such as integrated overcurrent protection, thermal shutdown, and undervoltage protection. Also, the DRV8874 allows for output current sensing, which can be used to detect motor stalls, changes in load conditions, or for the implementation of current-based control algorithms.

Lastly, for the DC motor, any low-voltage brushed DC motor within the driver and supply ratings can be used. However, heat sinking should be considered if operating near the DRV8874′s limit (6A), as it requires careful PCB layout design or additional heat dissipation measures. The PCB’s heat dissipation capacity at the driver’s nominal maximum current was not tested for the proposed device, as the maximum current of the chosen DC motor is well below this value (0.6 A stall current). Another recommended consideration is that the DC motor should come with an incremental encoder directly installed, to simplify wiring and device assembly (Table 3).

**Table 3:** Summary of the design files provided for reproducing the proposed hardware and software platform.

**Design file name**	**File type**	**Open-source license**	**Location of the file**
Schematic_DC_Motor.json	Circuit schematic	*CERN-OHL-P-2.0*	https://zenodo.org/records/19986592
PCB_DC_Motor.json	PCB layout	*CERN-OHL-P-2.0*	https://zenodo.org/records/19986592
Gerber_DC_Motor.zip	Gerber files	*CERN-OHL-P-2.0*	https://zenodo.org/records/19986592
Code.zip	Firmware/software	*MIT*	https://zenodo.org/records/19986592
DCMotor_base_svg.svg	CAD	*CERN-OHL-P-2.0*	https://zenodo.org/records/19986592
DCMotor_base_stl.stl	CAD	*CERN-OHL-P-2.0*	https://zenodo.org/records/19986592
DCMotor_base.step	CAD	*CERN-OHL-P-2.0*	https://zenodo.org/records/19986592

### 2.2 DC motor circuit schematic

**Fig. 1:**
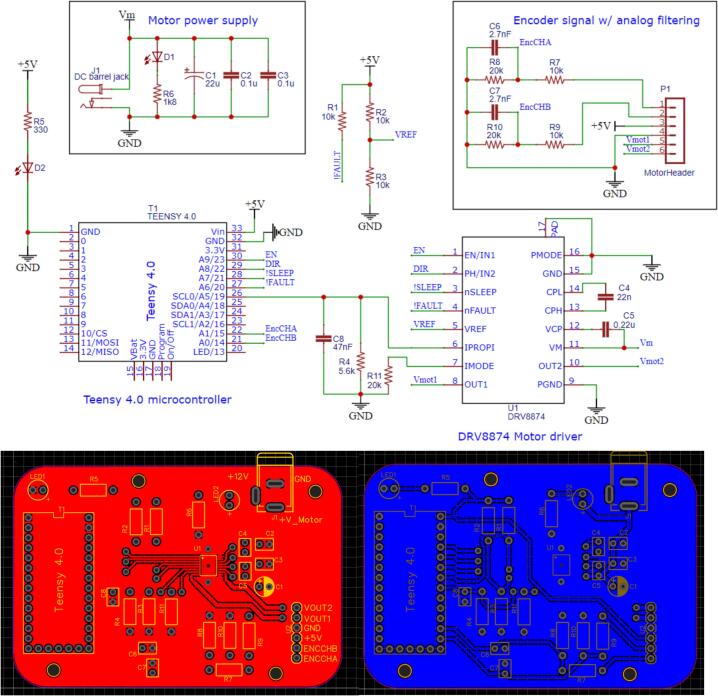
DC motor circuit schematic and PCB layout.

Fig. 1 depicts the schematic for interfacing the microcontroller, motor driver, and DC motor. The microcontroller receives its supply voltage via the USB connection to the host PC, with indicator LED D1 indicating when the microcontroller is powered on. The motor voltage (VM) is supplied through a DC barrel jack, connected to a standard 12 V power source. LED D2 serves as an indicator for motor supply availability. DRV8874′s *EN* (enable), *DIR* (direction) and $\overset{¯}{\mathrm{SLEEP}}$ I/O are connected directly to the microcontroller, while the $\overset{¯}{\mathrm{FAULT}}$ pin is connected via a pull-up resistor.

The IPROPI pin provides the current sense output, which, together with VREF, defines the current chopping threshold. Two additional configurable pins are IMODE and PMODE. PMODE is set to PH/EN mode (speed and direction control), and IMODE is configured to Quad-Level 2. In this mode, if the motor's current exceeds the trip threshold, the driver disables the MOSFETs and pulls the $\overset{¯}{\mathrm{FAULT}}$ pin low. After 2 ms, it retries automatically; if the overcurrent condition persists, the cycle repeats. The values used in this configuration depend on the nominal characteristics of the connected DC motor. A different motor may be used, but the circuit’s component values should be adjusted accordingly.

The DC motor connects through the P1 header, Vmot1 and Vmot2 being the driver power outputs to the motor, while the remaining four pins provide connections for the encoder signals: Enc ChA, Enc ChB, 5 V, and GND. Since the Teensy 4.0 is not 5 V tolerant, a voltage divider is used to reduce the encoder output levels to 3.3 V logic. Additionally, a capacitor is added to the voltage divider to act as a low-pass filter to mitigate noise from the encoder. The selected DC motor has a nominal speed of 300 RPM, and the encoder provides 240 pulses per revolution, yielding a maximum encoder frequency of 1.2 kHz. The filter's cutoff frequency is set to 3 kHz, by using a 2.7 nF capacitor (the cutoff frequency is calculated using Thevenin’s equivalent resistance on the capacitor’s terminals). Likewise, a RC filter ($f_{c}=600Hz)$ is added to the IPROPI output and is further filtered via software to get stable measurements.

### 2.3 Microcontroller embedded control firmware

The microcontroller embedded firmware was developed in C++ and deployed on a Teensy 4.0. The firmware was coded with a modular architecture separating hardware configuration, encoder feedback processing, signal generation, and digital control algorithms. The firmware is responsible for:

Reference Signal Generation: Supports diverse input signals such as Amplitude-Modulated Pseudo-Random Binary Sequence (APRBS), sinusoidal, square, and chirp signals for comprehensive system identification.
Control Algorithms: Implements a customizable PID controller (incremental and positional PID) with anti-windup and low-pass-filtered derivative, a general-purpose digital controller defined explicitly by difference equations. While commercial platforms can also support custom controller implementation through their respective software ecosystems, the proposed platform places explicit coefficient-level digital-controller implementation at the center of the student workflow within an open and modifiable environment.
Communication and Data Logging: Provides real-time serial communication, allowing interactive parameter tuning and immediate feedback, significantly enhancing the educational value through iterative experimentation and visualization.

### 2.4 Graphical user interface

**Fig. 2:**
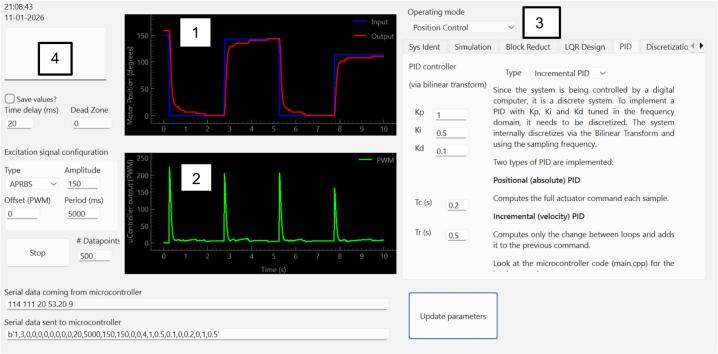
GUI position control application example. It includes 1: input reference and measured values, 2: output value (i.e. the output from the microcontroller to the dc motor), 3: operation mode: can be set to system identification, speed control and position control and 4: configurable reference signals and control parameters.

A Python-based graphical user interface (GUI) was developed with PyQt and PyQtGraph and complements the hardware by offering an interactive educational environment (see Fig. 2). This interactivity transforms the hardware into a tool for active and experiential learning, allowing students to directly observe the impact of their design decisions in real-time. Its main features include:

Real-time visualization and logging: dynamic plots of reference, measured speed, current, loop period, and PWM output, together with continuous data capture for further analysis.
Interactive parameter configuration: real-time adjustment of excitation signals, PID parameters, and discrete controller coefficients without interrupting system operation.
Modeling and analysis tools: empirical system identification from measured data, time-response simulation, block reduction, and continuous- and discrete-time control analysis within the same environment.
Controller design support: interactive continuous-time PID tuning, plant discretization, discrete-time controller design, and export of the resulting parameters to the embedded implementation.

It is worth mentioning that the GUI is protocol-driven and can be reused with other plants by adapting the microcontroller’s firmware to stream the expected variables in the required order.

## 3 Design files summary

The circuit schematic, the PCB layout and the Gerber files were generated using EasyEDA. Code was developed in C++ and Python. CAD files were designed using Autodesk Fusion 360 and exported to commonly used, non-proprietary file formats.

1. Schematic_DC_Motor.json: schematic for the DC motor controller circuit, can be loaded directly to EasyEDA.
2. PCB_DC_Motor.json: PCB layout for the provided schematic, can be loaded directly to EasyEDA.
3. Gerber_DC_Motor.zip: files for the fabrication of the PCB in a standardized format, used by PCB manufacturing services.
4. Code.zip: ZIP file containing necessary files for the hardware. Can be imported directly as a project using PlatformIO or other IDE. Its main files are:
  • main.cpp: microcontroller embedded firmware for the DC motor control.
  • GUI.py: user interface for displaying and modifying the controller parameters.
  • QtDesignerGUI.ui: Qt Designer graphical user interface shell with drag and drop controls and displays. Is a dependency of GUI.py.
5. DCMotor_base_svg: base for the motor if using a laser cutting machine.
6. DCMotor_base_stl: base for the motor if using a 3D printer.
7. DCMotor_base.step: editable file for the motor base.

## 4 Bill of materials summary

Table 4.

**Table 4:** Bill of materials for the proposed DC motor control educational kit.

**PCB Board**						
**Designator**	**Component**	**Number**	**Cost per unit − USD**	**Total cost − USD**	**Source of materials**	**Material type**
C1	Capacitor, electrolytic − 22 u	1	0.16	0.16	Mouser	Metal
C2, C3	Capacitor - 100*n*	2	0.87	1.74	Mouser	Ceramic
C4	Capacitor - 22*n*	1	0.35	0.35	Mouser	Ceramic
C5	Capacitor - 220*n*	1	0.35	0.35	Mouser	Ceramic
C6, C7	Capacitor - 2.7*n*	2	0.3	0.6	Mouser	Ceramic
C8	Capacitor - 47*n*	1	0.3	0.3	Mouser	Ceramic
D1, D2	LED diode	2	0.24	0.48	Mouser	Semiconductor
DC barrel jack	DC barrel jack	1	1.02	1.02	Mouser	Composite
DRV8874	Motor driver	1	3.05	3.05	Mouser	Semiconductor
P1	Header 1x6	1	0.34	0.34	Mouser	Composite
R1, R2, R3, R7, R9	Resistor − 10 k	5	0.43	2.15	Mouser	Ceramic
R4	Resistor − 5.6 k	1	0.65	0.65	Mouser	Ceramic
R5	Resistor − 330	1	0.7	0.7	Mouser	Ceramic
R6	Resistor − 1 k8	1	0.65	0.65	Mouser	Ceramic
R8, R10, R11	Resistor − 20 k	3	0.53	1.59	Mouser	Ceramic
Teensy 4.0	Microcontroller	1	23.8	23.8	Mouser	Composite
						
**Other components**						
NA	DC Motor w/ encoder	1	21.99	21.99	Amazon	Metal
NA	DC Motor mounting bracket	1	7.99	7.99	Amazon	Metal
NA	Micro USB to USB cable	1	10.38	10.38	Amazon	Composite
NA	12 V Power Supply	1	11.99	11.99	Amazon	Composite

## 5 Build instructions

**Fig. 3:**
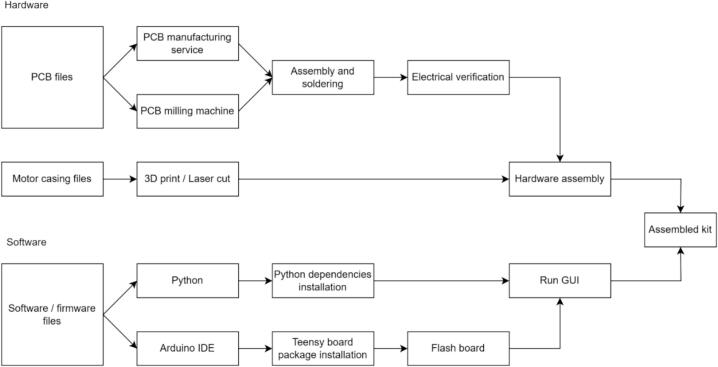
Procedure for reproducing the proposed kit from the released open-source resources, including hardware fabrication and software installation and flashing.

A general workflow for the fabrication of the proposed device is described in Fig. 3, whereas the following subsections describe in detail each procedure.

### 5.1 PCB manufacturing

**Fig. 4:**
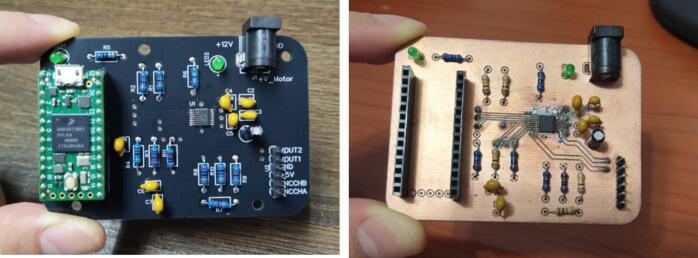
PCB assembled using a prototyping service and a maker-lab CNC milling machine.

The printed circuit board (PCB) for the DC motor module can be fabricated using the Gerber_DC_Motor.zip. The design follows standard two-layer PCB manufacturing practices and can be produced either in a maker lab or by any prototyping service (e.g., JLCPCB, PCBWay, or OSH Park). The PCB layout (PCB_DC_Motor.json) considers design-for-manufacturing and design-for-testing practices (see Fig. 4). Since the focus of this hardware is for students being able to fabricate it from scratch, the PCB uses mainly through-hole components in a spacious PCB layout, so that they can be easily soldered manually. Additionally, the PCB layout has exposed vias, which is a good practice for signal testing and debugging.

### 5.2 Soldering and assembly

Once the PCB is fabricated, solder the components following the schematic (Schematic_DC_Motor.json) and the Bill of Materials (Section 4). We strongly recommend soldering the DRV8874 first, then continue with low-profile components (resistors, capacitors) before moving to larger parts (LEDs, headers, and connectors). The DRV8874 relies on the thermal pad for heat sinking and requires careful soldering with a hot-air rework station or reflow oven, or its current capacity could be significantly degraded. A soldering iron is not recommended for this component. All other components are through-hole, which can be soldered manually or using solder paste and hot-air reflow. After assembly, visually inspect all joints for shorts or cold soldering and verify continuity using a multimeter before connecting power.

### 5.3 PCB/Motor support

**Fig. 5:**
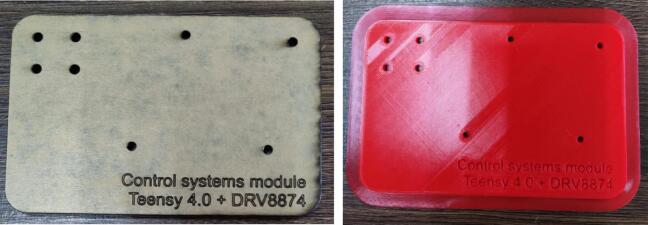
Proposed hardware mounting base. Option a: on acrylic via laser cutting machine, option b: via 3D printer.

A base for mounting the PCB and the DC motor to a single piece is provided in three different formats, which can be used depending on the availability of manufacturing hardware. The DCMotor_base_svg.svg corresponds to the required file for laser cutting the base (e.g. acrylic), the DCMotor_base_stl.stl for 3D printing (see Fig. 5), and if an editable version of this file is required, use the DCMotor_base_step.stp file. The DC motor and the PCB board can be attached to the base using screws and brass inserts.

## 6 Operation instructions

This section gives step-by-step directions to install the software, load the microcontroller firmware, and operate the hardware.

### 6.1 Install the software

Download Code.zip from the repository and extract the following files: main.cpp, GUI.py and QtDesignerGUI.ui.

### 6.2 Installing the microcontroller software

Install Arduino IDE and the Teensy add-on (Teensyduino).
Open main.cpp and copy its contents to a new Arduino sketch.
Set Board: Teensy 4.0, USB Type: Serial.
Connect the Teensy 4.0 via USB and click Upload.After uploading, the board resets and waits for serial commands.

### 6.3 Installing dependencies for the GUI

**Fig. 6:**
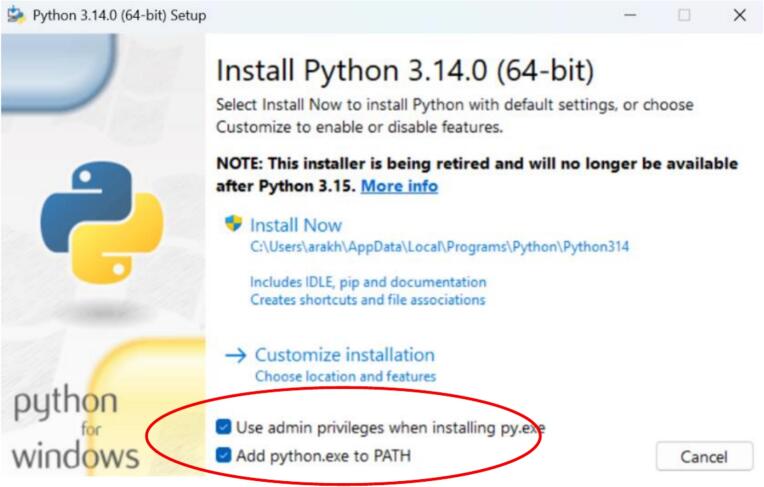
Check admin privileges and add python.exe to path when installing.
After python is installed, open a Windows PowerShell and install the GUI dependencies with the following command:

Install python.
o We strongly recommend checking admin privileges and adding python.exe to PATH during installation (see Fig. 6).

python −m pip install pyqt6 pyqtgraph numpy pandas scipy control pyserial.

### 6.4 Connect the hardware

**Fig. 7:**
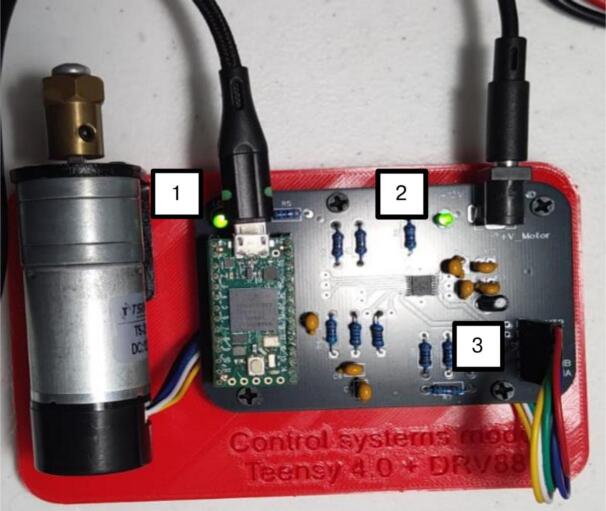
Teensy’s micro-USB connector. 1: LED1 indicates connectivity between the microcontroller and the host PC. 2: LED2 indicates the 12 V power supply is connected to the board. 3: motor leads.

Power on the motor/encoder (see Fig. 7).

• Connect a regulated 12 V DC supply to the board’s barrel jack.
• Connect the motor leads to the DRV8874 motor output header.
• Connect encoder A, B, VCC, GND to the encoder header.
  o Be careful when connecting the motor leads, as swapping the motor/encoder connectors will damage the encoder.
• Connect the Teensy 4.0 to the host PC via USB.
• Ensure no other serial terminal is open (only the GUI should use the port).

### 6.5 Run the system

Open a Windows PowerShell terminal and change directories (via *cd* command) to the folder where the GUI.py script is held.
Start the GUI.py by typing “python GUI.py”, select the COM port if prompted.

The GUI auto-detects common boards. If your board is not automatically detected, the GUI displays a COM port selection dialog box.

### 6.6 Notes and safety considerations

**Fig. 8:**
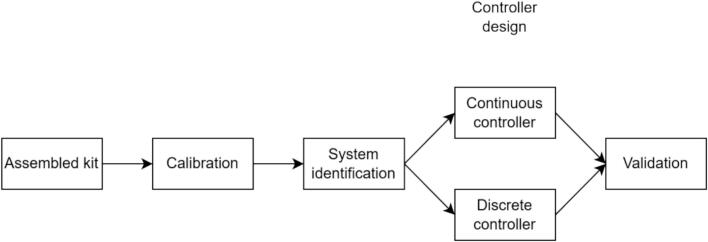
Control design workflow for the proposed hardware.

• Before the first power-up, use a current-limited supply (or fuse) and check for shorts with a multimeter.
• Verify the motor spins freely and is firmly mounted.
• Operate at ≤ 12 V DC. Keep hands, hair, and loose items away from the shaft (Fig. 8).

## 7 Validation and characterization

To demonstrate the proposed hardware’s workflow, we first characterize the plant to define the operating range over which a linear time-invariant (LTI) model is appropriate. We then identify a plant model from data acquired with the GUI, use the same environment for controller tuning and discrete-time controller design, and finally implement both continuous-time and discrete-time speed controllers on the platform to compare simulation results with the measured experimental response.

### 7.1 Sensor calibration

**Fig. 9:**
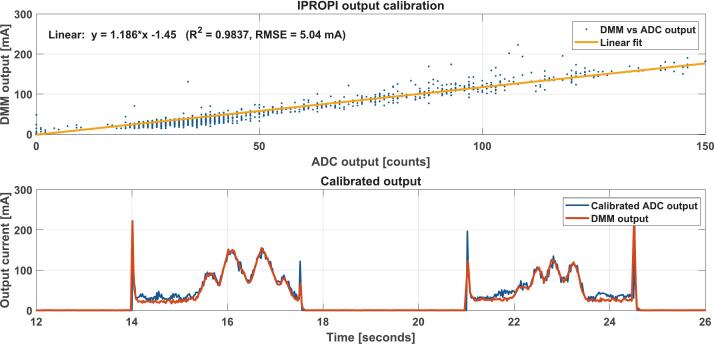
Output current calibration data and results. Top: Teensy’s Analog-to-Digital Converter (ADC) output vs Siglent’s SDM3045X Digital Multimeter (DMM) output. Bottom: Current measurements via the DRV8874 IPROPI pin after calibration. Output current was varied by manually braking the motor to obtain different load conditions.

Before proceeding with the control system implementation, we first calibrate the motor current measurement using the DRV8874 IPROPI output. Calibration was performed with a Siglent SDM3045X digital multimeter (Shenzhen, China) in fast acquisition mode with filtering disabled. Because the multimeter and the proposed device use different sampling times, interpolation was used to align the multimeter data with the data acquired via the GUI. The calibration points were obtained by manually braking the motor to cover a variety of load conditions. The ADC readings from the Teensy 4.0 were then compared against the synchronized DMM current measurements to obtain the calibration relationship used to convert the IPROPI signal into current values. The calibration results can be seen in Fig. 9.

### 7.2 System identification

**Fig. 10:**
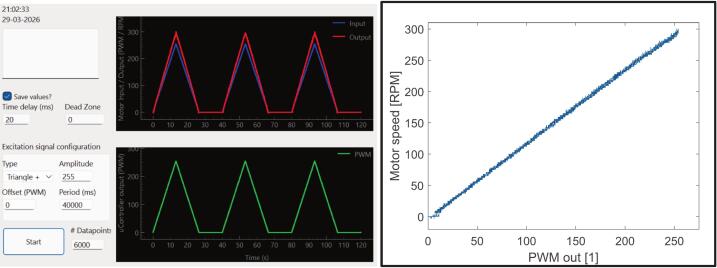
Static nonlinearities measurement by applying a slow triangular command signal to the motor.

As part of the control design workflow, we measure static nonlinearities (dead zone, hysteresis, and saturation) by applying a triangular command signal ranging between 0 and 255. From the resulting input–output map, we determine the dead-zone thresholds and the operating region over which the motor response is approximately linear. Samples within the dead zone and near actuator saturation are removed from the dataset used for model fitting and motor operation. As shown in Fig. 10, the proposed system exhibits an almost linear speed to PWM relationship, with a dead zone of approximately 16 PWM counts and no apparent saturation nor hysteresis in the measured range. This is mostly due to the DRV8874′s charge pump and low $R_{\mathrm{DS}(on)}$ [30].

**Fig. 11:**
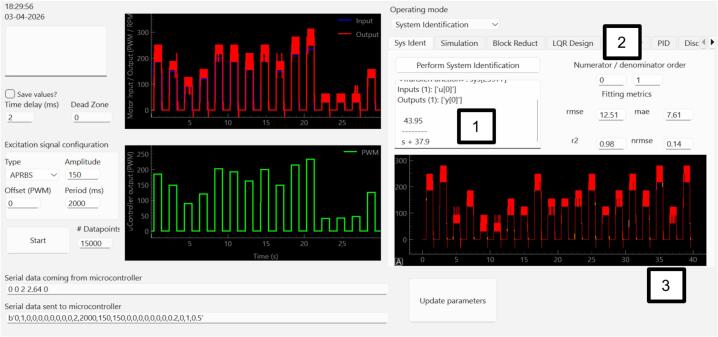
DC motor experimental system identification using 0 zeros and 1 pole. 1: System identification result. 2: Numerator/denominator order. 3: Graphical representation of the simulated and measured data overlap.

We then identify the plant model using data acquired with the GUI. For this purpose, an APRBS input is applied, generating random PWM command values from 30, to remain above the dead zone identified earlier, up to 255 [31]. During the experiment, the GUI records the applied PWM input, the measured motor speed, and the measured loop period. For identification, the user first selects the numerator and denominator orders of the transfer-function model in the GUI. For that fixed model structure, the time base is reconstructed from the measured sampling intervals, and the unknown numerator and denominator coefficients are estimated by nonlinear least squares using SciPy’s curve_fit function [32], with the leading denominator coefficient fixed to 1. At each iteration, the candidate transfer function is simulated using the same measured PWM input employed in the experiment, and the fit is obtained by minimizing the time-domain squared error between the measured and simulated motor-speed outputs. The identified response is then compared against the measured response in the GUI, while fit metrics such as RMSE, MAE, $R^{2}$, and NRMSE are reported to help the user compare different model orders and select the most appropriate structure (see Fig. 11). The resulting model is then handled and analyzed within the GUI using python-control [33], [34]. Readers interested in more specialized open-source identification workflows may also consider packages such as SysIdentPy [35].

It is worth highlighting that the identified parameters are sensitive to the selected sampling period. This is an expected behavior because the discrete dataset must capture the motor’s transient behavior with enough time resolution. Shorter loop periods lead to more reliable identification of the motor dynamics.

### 7.3 Continuous-time controller design

After identifying the plant, the next step is controller design. Controller design can be done either by manual calculations or via software and both methods should be covered in an educational scenario. For this reason, the proposed GUI includes an interactive continuous-time PID tuning module that allows the user to select the controller structure, preview the closed-loop step response, and then export the resulting gains to the PID deployment tab for implementation.

The identified transfer function from section 7.2 is given by Eq. (1).

(1) $$G_{\mathrm{plant}}\left(s\right)=\frac{43.95}{s+37.9}$$

Using the PID Tuner tab, the identified plant can be loaded directly from the system identification tab. The user then selects the controller type (P, PI, PD, or PID) and adjusts the desired closed-loop behavior using two sliders: Response speed and Transient behavior. For each slider position, the GUI evaluates the corresponding continuous-time controller gains and updates the simulated closed-loop step response in real time. The larger tuner window also provides the associated controller gains and closed-loop performance metrics, allowing the user to inspect the design before deployment.

**Fig. 12:**
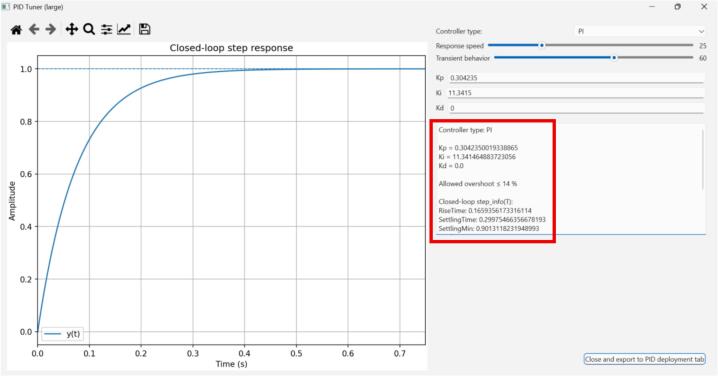
Continuous-time PI controller design via the GUI’s extended PID Tuner view. For the selected design objective (approximately 300 ms settling time for the identified plant), the tuner yields $K_{p}=0.3042$ and $K_{i}=11.3415$.

**Fig. 13:**
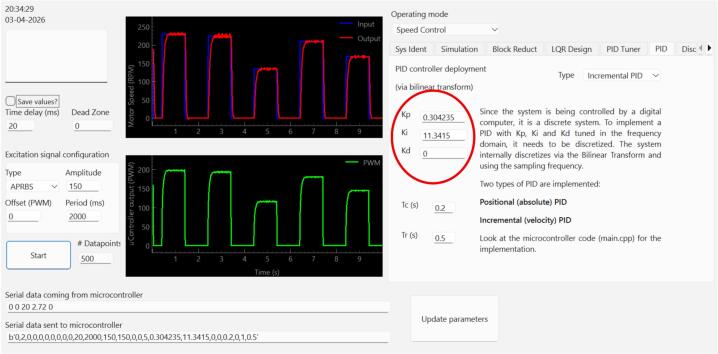
Continuous-time PI controller deployment in the PID tab of the GUI. The continuous-time gains are entered directly, while the embedded implementation internally discretizes them through the bilinear transform. Data containing the input reference, measured speed, measured loop period, measured current, and PWM output can be saved for further analysis using the Save values? checkbox.

For this example, we chose a 300 ms settling time as the design objective. To avoid degrading the system’s performance due to discretization, the sampling time should be 10 to 20 times faster than the dominant closed-loop dynamics (settling time). Thus, for a 300 ms settling time, we chose 20 ms as the sampling time. The desired transient response can be achieved with a PI speed controller as shown in Fig. 12. The implementation results can be seen in Fig. 13 and Fig. 15.

**Fig. 14:**
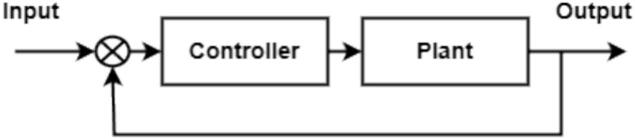
General architecture of the implemented system. For this example, the controller block represents the tuned PI controller, whereas the plant block represents the identified transfer function from Eq. (1).

To further connect controller tuning with analysis, the GUI also includes a Control Console tab, which allows the user to combine transfer functions and evaluate the resulting closed-loop model directly within the same environment. For the controller designed in this example, the identified plant is combined with the selected PI controller under unity feedback to obtain the equivalent closed-loop transfer function used for simulation and inspection before deployment (see Fig. 14). This makes it possible to verify the overall response of the compensated system from the same GUI, without relying on external software.

**Fig. 15:**
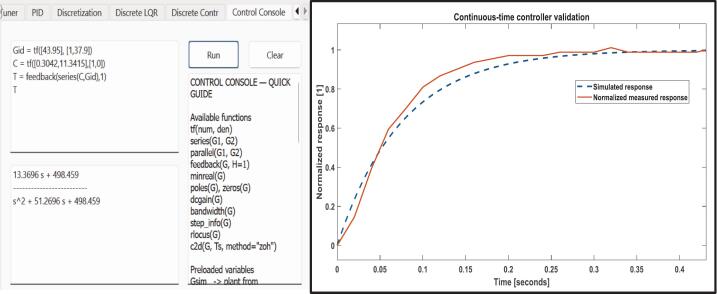
Comparison between the simulated output and the normalized measured output of the system. The simulated output equation was obtained by combining the identified transfer function (Eq. (1) and the PI controller using the Control Console tab of the GUI. The measured output was obtained by saving the output data using the Save values? checkbox of the GUI.

The equivalent closed-loop transfer function can be obtained in the Control Console tab by combining the identified plant from Eq. (1) with the PI controller from Fig. 12 under unity negative feedback, using the series and feedback.

For the PI controller,

(2) $$C\left(s\right)=K_{p}+\frac{K_{i}}{s}=0.3042+\frac{11.3415}{s}$$

The resulting closed-loop model is then used for simulation and compared against the normalized measured response, as shown in Fig. 15.

### 7.4 Discrete-time controller design

**Fig. 16:**
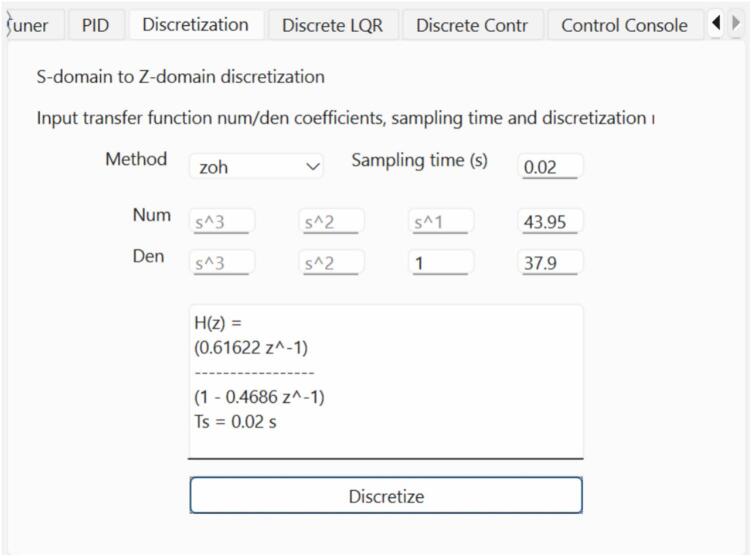
GUI Discretization tab for discretizing the plant's identified transfer function to the discrete domain using the Zero-Order Hold (ZOH) method.

**Fig. 17:**
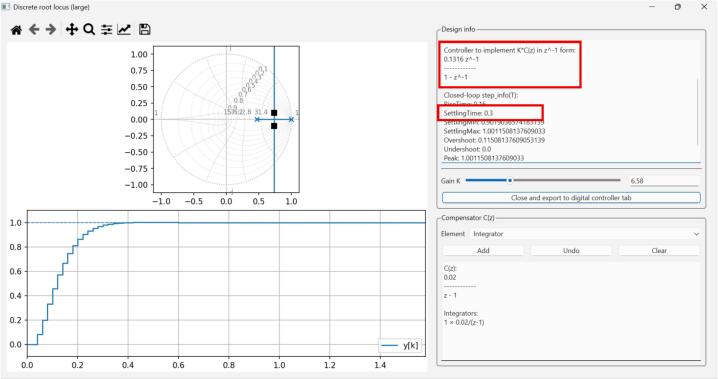
GUI Discrete Root Locus Design tab. The interface displays the unit circle and the time response for the plant with the controller. For this example, the target is a settling time of 0.3 s. The interface also shows the discrete controller needed for achieving the target behavior.

A discrete controller can be designed either by discretizing the plant model and designing the controller in the z-domain or by designing the controller in the s-domain and then discretizing the controller. We chose the former to demonstrate the discrete controller implementation using the GUI (Fig. 16 and Fig. 17).

To discretize the plant, we use the identified plant model obtained in section 7.2 and discretize using the zero-order hold method with a sample time $T_{s}=0.02s$ (designing for 300 ms settling time as in the previous example).

This results in Eq. (3):

(3) $$G_{\mathrm{plant}}\left(z\right)=\frac{0.61622}{z-0.4686}$$

We then import the discretized plant model to the Discrete Root Locus Design tab (Discrete LQR) of the GUI. Being a type 0 system, we aim to correct the steady-state error by adding a discrete integrator. For error correction and 300 ms settling time, the controller required is given by Eq. (4).

(4) $$C_{\mathrm{controller}}\left(z\right)=\frac{0.1316}{z-1}=\frac{{0.1316z}^{-1}}{1-z^{-1}}$$

**Fig. 18:**
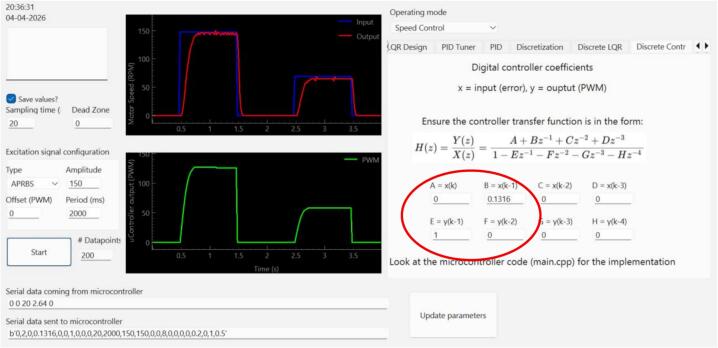
Discrete-time speed controller implementation using the Discrete Controller tab of the GUI. Note that the coefficients should be in the correct form for a correct implementation.

We use the resulting coefficients for implementing the discrete controller in the Discrete Controller tab of the GUI (Fig. 18).

### 7.5 Data streaming rate and loop control period

**Fig. 19:**
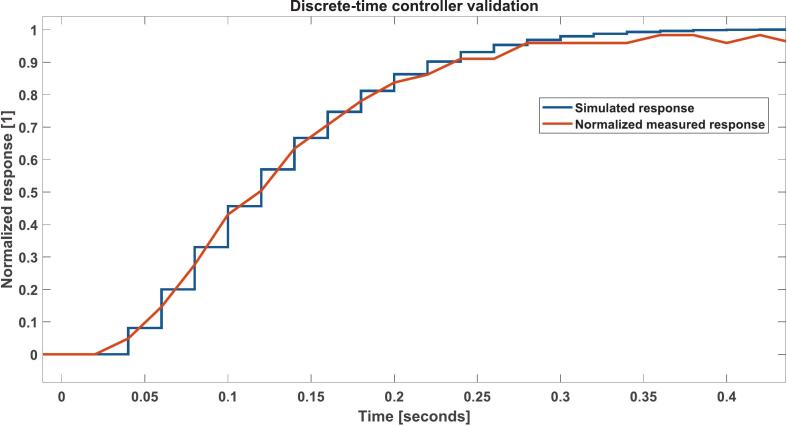
Discrete-time speed controller validation. The simulated response was obtained by simulating a step response of the equivalent, closed-loop transfer function and the measured response was obtained by checking the Save values? checkbox of the GUI.

The microcontroller’s embedded firmware executes a time-triggered loop with a user-configurable period set from the GUI (“Sampling time (ms)”). The same loop also streams reference, measured speed, current, measured loop period, and PWM output over USB serial, so the streaming and visualization update rate is coupled to the embedded loop period (Fig. 19).

### 7.6 Thermal considerations

**Fig. 20:**
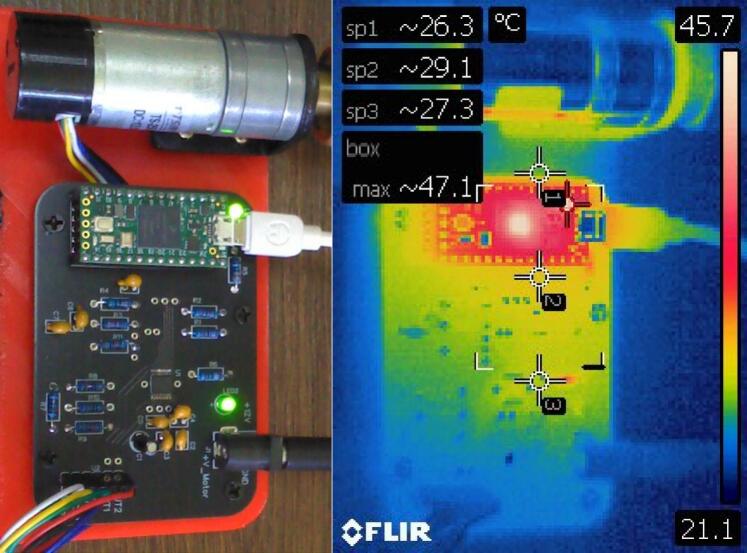
Thermal image of the proposed hardware after 1 h of continuous operation at nominal voltage (no load).

Even if the DRV8874 can handle peak currents of up to 6 A while limiting the output current via automatic current regulation, the PCB layout requires careful design and attention to component placement. For that reason, the hardware presented in this work implements the layout recommendations given by the manufacturer [30]. However, even if the PCB layout is correct, an incorrect soldering technique could lead to insufficient heat sinking. To verify correct heat sinking, the motor was operated continuously during one hour at nominal output voltage (12 V or 255 PWM) under no-load condition and external temperature was measured using a FLIR E4 Thermal Camera (Teledyne FLIR, OR United States), see Fig. 20.

As expected, the highest temperature point corresponds to the microcontroller, whereas the rest of the board, and especially the DRV8874, has negligible temperature increase.

### 7.7 Capabilities and limitations

#### 7.7.1 Capabilities

• Interactive GUI for visualization, parameter tuning, and data logging (reference, measured speed, current, loop period, PWM) with a soft real-time control loop embedded in the microcontroller.
• Supports continuous-time PID tuning and arbitrary discrete-time controllers via user-defined difference-equation coefficients.
• Built-in workflow for characterizing actuator non-idealities (dead zone, hysteresis, and saturation) and using filtered encoder/current measurements for experiments.
• Design is open and modifiable (firmware + PCB + GUI), so users can add new signal types, controllers, sensors, or plants.

#### 7.7.2 Limitations

• Plant is based on a brushed DC motor, which generally have a shorter service life compared to other motor types due to wearing of the brushes.
• Streaming and plot update rates are coupled to the microcontroller’s firmware loop period and may be limited by GUI plotting overhead (especially when trying very low sampling times on slower PCs).
• Correct operation near the driver’s maximum output current strongly depends on the PCB’s thermal design and soldering quality.

## Ethics statements

No human or animal studies were conducted in this work.

## CRediT authorship contribution statement

**Alejandro Von Chong:** Writing – original draft, Validation, Software, Methodology, Conceptualization. **Salvador Vargas:** Writing – review & editing, Visualization. **Jean-François Duhé:** Supervision. **Dorindo Cárdenas:** Writing – review & editing.

## Declaration of competing interest

The authors declare that they have no known competing financial interests or personal relationships that could have appeared to influence the work reported in this paper.
